# Editorial: Soundscape, well-being and mental health during/after the COVID-19 pandemic

**DOI:** 10.3389/fpsyg.2023.1340207

**Published:** 2023-12-11

**Authors:** Yue Wu, Jian Kang, Fangfang Liu, Hui Xie, Stephen Lau

**Affiliations:** ^1^Key Laboratory of Cold Region Urban and Rural Human Settlement Environment Science and Technology, School of Architecture, Harbin Institute of Technology, Harbin, China; ^2^Institute for Environmental Design and Engineering, The Bartlett, University College London, London, United Kingdom; ^3^School of Architecture, Chongqing University, Chongqing, China; ^4^Faculty of Architecture, The University of Hong Kong, Hong Kong, Hong Kong SAR, China

**Keywords:** soundscape, wellbeing, mental health, COVID-19 pandemic, human perception

Coronavirus disease (COVID-19), a new coronavirus pneumonia, has been the most devastating and lethal global public health catastrophe over the last 100 years. On May 5, 2023, the World Health Organization officially declared “COVID-19 Global Health Emergency is over.” Several countries adopted strict lockdowns in families and communities as the primary tactic to combat the pandemic. These actions have profoundly changed the acoustic environment and urban soundscape, and impacted residents' sound perception. Simultaneously, these regulations have exacerbated residents' psychological distress and negatively impacted their physical and mental health (Zhou et al., 2020). COVID-19 has caused and continues to cause deep scars worldwide.

Soundscape perception is likely to be influenced by a combination of factors (Jiang et al., 2022; Liu et al., 2022), and countless individual and environmental attributes interact to shape an individual's attitudes and acoustic environmental changes during the COVID-19 pandemic (Aumond et al., 2022). As research continues to evolve, a more comprehensive understanding of specific changes in the acoustic environment and urban soundscapes has been explored, and scholars from highly diverse disciplines have sought to provide insights into their potential influence on their potential influence on human perception, wellbeing, and mental health (Alonso et al., 2022; Ge et al., 2023) in this consequential and complex domain.

This Research Topic intends to present how this global health emergency has changed the acoustic environment and soundscape perception, affected residents' mental health and wellbeing, and described targeted interventions and preventative measures to help those affected. These contributions demonstrate the breadth of interest in this topic, and seven articles by 27 authors have been published.

A number of papers focused on the acoustic environment, either by comparing the changes before and after the lockdown or by investigating interventions to support people. Sound environment in an urban apartment building was investigated by Yang and Kang investigated the sound environment by monitoring noise in a 14-story apartment building surrounded and shielded by other buildings in a typical urban community during and after the lockdown. The results indicated that the A-weighted sound pressure level (LAeq) of all 14 floors after the lockdown was higher than that during the lockdown. Park et al., conducted a hospital-based investigation of Korean patients and staff members, given that they were particularly vulnerable to emotional issues during the COVID-19 pandemic. Satisfaction with the indoor acoustic environment and odor were significant predictors how patients perceived the indoor environment to be helpful in their recovery from COVID-19. These insights can help policymakers and hospital administrators to develop strategies to create hospital environments that meet the requirements of both groups. Torresin et al. conducted a survey comprising 464 respondents living in London during the COVID-19 lockdown to identify the characteristics of an “ideal” indoor soundscape. The ideal soundscape was described as a quiet, well-sound insulated environment, which guarantees access to positive sounds (i.e., natural sounds, music, and urban background), thus resulting in privacy, intimacy, and a place to express themselves without noise-related constraints.

Some articles in this Research Topic have examined the relationship between environmental indices and human perception during the COVID-19 pandemic. The study by Ren et al. utilized five indicators (willingness to walk, relaxation, safety, beauty, and comprehensive comfort) to measure the environmental health of pedestrian streets with traffic noise. Yu et al. also used a cross sound–light sensory channel to study the interaction of sound–light pollution in urban residential environments at night, and found that visual elements can change individuals' perception of sound, but the tolerance limit of participants was not reduced due to the superposition of two intrusive variables.

Other articles in this Research Topic have focused on the role of the acoustic environmental through measurement and subjective evaluation. The study by Mu and Kang is about the Dining comfort in elderly care facility dining rooms before and after the COVID-19 pandemic, by measuring the physical environment of the facilities, analyzing dining behavior, and surveying the elderly residents. Qu et al. conducted field survey to identify the soundscape and non-acoustic factors related to aircraft noise evaluation. The findings implied the moderating effects of subjective factors and the restorative effects of natural sounds, which could inform aircraft noise control and community consultation strategies by protecting vulnerable populations and creating community soundscape facilities.

This Research Topic of papers makes a unique contribution to the soundscape literature on the COVID-19, advancing our understanding of the wide-ranging effects of the pandemic. These diverse studies further illuminate our understanding of the restorative effect of soundscapes after people have experienced the pandemic, providing valuable insights for both scholars and practitioners.

## Author contributions

YW: Writing—original draft. JK: Writing—review & editing. FL: Writing—review & editing. HX: Writing—review & editing. SL: Writing—review & editing.
